# Contingency management for smoking cessation among individuals with type 2 diabetes: protocol for a multi-center randomized controlled feasibility trial

**DOI:** 10.1186/s40814-020-00629-7

**Published:** 2020-06-11

**Authors:** Sydney A. Martinez, Samantha L. Quaife, Afsheen Hasan, Kathryn A. McMillan, Laura A. Beebe, Fiona Muirhead

**Affiliations:** 1 Department of Biostatistics and Epidemiology, Hudson College of Public Health, University of Oklahoma Health Sciences Center, 801 NE 13th Street, Oklahoma City, OK 73104 USA; 2 Research Department of Behavioural Science and Health, University College London, Gower Street, London, WC1E 6BT UK; 3 Digital Health and Wellness Group, Computer and Information Sciences, University of Strathclyde, Livingstone Tower Building, Glasgow, G1 1QE UK; 4 Physical Activity for Health, School of Psychological Sciences and Health, University of Strathclyde, Graham Hills Building, 50 George Street, Glasgow, G1 1QE UK

**Keywords:** Smoking cessation, Diabetes, Financial incentives, Contingency management, mhealth

## Abstract

**Background:**

Individuals with type 2 diabetes (T2D) who smoke are at increased risk for many types of cancers as well as an accelerated progression of microvascular and macrovascular complications. Smoking cessation is recommended as a standard treatment for T2D; however, individuals with T2D are faced with competing lifestyle changes. Glycemic and blood pressure control often take precedence over smoking cessation, and patients are often unmotivated to quit. Contingency management in combination with standard smoking cessation treatment has been demonstrated to improve cessation outcomes in various populations. The purpose of this randomized controlled feasibility trial is to explore the feasibility of contingency management and biochemical verification using a remote smartphone-based carbon monoxide monitor for smoking cessation among individuals with T2D.

**Methods:**

A three-arm, randomized controlled feasibility trial will be conducted in two study sites that include the USA and UK. We will recruit 60 participants who will each receive usual care smoking cessation treatment (counseling and nicotine replacement therapy) and be randomized to a short term incentives (6 weeks), long term incentives (12 weeks), or no incentives (control) group. Participants will receive a smartphone and carbon monoxide monitor to complete daily remote assessments throughout the 12 weeks and will complete an exit interview at the end of the study. The primary outcomes for this feasibility study include completion of the protocol and proportion of daily assessments completed. Secondary outcomes include recruitment measures, acceptability, and smoking abstinence.

**Discussion:**

We will explore the feasibility of recruiting smokers with T2D and their engagement in the program, particularly related to the use of the remote biochemical verification and smartphone application. In addition, we will evaluate the intervention content, study procedures, data collection methods, and follow-up and will qualitatively assess the participants’ acceptability of the program. The results of this study will inform the design of a larger trial to test the efficacy of the contingency management program for improving smoking cessation outcomes among individuals with T2D.

**Trial registration:**

This randomized controlled feasibility trial has been registered at ClinicalTrials.gov with an ID NCT03527667 on May 4, 2018.

## Background

Type 2 diabetes (T2D) and cigarette smoking are major causes of morbidity and mortality in the USA (US) and United Kingdom (UK) [1–5], and research indicates there is a complex relationship between these two risk factors [6–10]. Smoking is a major behavioral risk factor for many types of cancer, is associated with insulin resistance, and was listed as a causal factor for T2D in the 2014 Surgeon General’s Report [4, 6, 11]. The harms of smoking are particularly profound among individuals with T2D who continue to smoke, with an accelerated progression of microvascular and macrovascular complications and an increased mortality risk [7, 8, 12]. Individuals with T2D are also independently at increased risk for many cancers, although the link between T2D and cancer is currently not well understood [13, 14]. Evidence suggests biological mechanisms along with increased prevalence of behavioral risk factors play a key role [6, 14–16]. Despite the heightened risk for cardiovascular disease, cancer, and diabetes-related complications, many patients with T2D continue to smoke. As the prevalence of T2D in the US and UK population is expected to double in the next three decades, it is essential that we understand the impact of smoking on diabetes and health outcomes and identify ways to effectively integrate smoking cessation into diabetes care [5, 17].

As standard care, those diagnosed with diabetes who smoke are recommended by the American Diabetes Association and the UK’s National Institute of Health and Care Excellence to stop smoking; however, patients with T2D and their providers are often inundated with other challenging lifestyle changes and disease management strategies [18, 19]. Glycemic and blood pressure control through dietary changes often take precedence over cessation, and patients often feel unmotivated or are unable to quit smoking [19]. Due to the complex relationship between T2D and smoking and the large number of competing lifestyle changes recommended at diagnosis, smokers with T2D may benefit from a program that incentivizes smoking cessation.

Providing patients with tangible rewards to reinforce positive behaviors, such as smoking abstinence, has been proven effective during treatment in substance abuse programs, smoking cessation programs among pregnant women unwilling or unable to quit, and other population subgroups [1, 20–24]. Contingency management, or the exchange of a reward contingent upon verified smoking abstinence, in combination with standard smoking cessation treatment, has been demonstrated to improve cessation outcomes in various populations [1, 20, 24–29]. Few studies have examined the effects of a contingency management program or financial incentives for smoking cessation among individuals with chronic disease [30]. Through a retrospective analysis of randomized trials, Walter and Petry demonstrated that participants with diabetes responded well to contingency management as a treatment for substance abuse and that the effect of the incentives was greater than for individuals without diabetes [31]. Other studies among patients with diabetes have focused on contingency management for diabetes medication adherence [32, 33]. Despite the heightened risks of smoking-related outcomes among individuals with T2D, to our knowledge, contingency management has not been tested in this population as a smoking cessation intervention. It is unclear whether a contingency management program would be feasible and acceptable in this population.

The need to biochemically verify smoking abstinence daily creates a challenge for many contingency management programs for smoking cessation. Smoking abstinence is primarily verified through saliva, urine, or carbon monoxide (CO) assessments, but challenges around the costs, difficulty of administration, and logistics of daily testing has been a limiting factor in contingency management studies [26, 34]. Researchers have had to choose whether to place the burden of many in-person visits on the participants or incur significant data collection costs through daily home visits by study coordinators. With the rapid expansion and development of mobile health (mHealth) technology, there are now smartphone-based remote CO sensors that can be distributed to each research participant for daily verification of smoking abstinence [27, 34–36]. In addition, the combined use of a smartphone sensor and application (app) provides the opportunity for daily assessment of urges to smoke, motivation to quit, tobacco use, and medication adherence. If ultimately proven feasible and effective, this remote smartphone approach also provides a scalable and cost-effective means of delivering this intervention in a healthcare setting.

## Study aims and hypotheses

The research objectives and trial design were conceived and developed through a unique multidisciplinary and international collaboration formed during a knowledge integration sandpit event hosted by the US National Cancer Institute and Cancer Research UK. This was a residential workshop bringing together investigators from the UK and the US across different sectors and disciplines (e.g., academia, technology, epidemiology, psychology, community) to integrate knowledge about behavioral cancer risk factors, form new networks, and develop innovative research grant proposals in cancer prevention. Through this, we designed a randomized controlled feasibility trial to explore the acceptability and feasibility of a contingency management program for smoking cessation among smokers with T2D previously unable to quit. This pilot study aims to gather data in both the US and UK context related to the feasibility of conducting a contingency incentive-based program with remote biochemical verification using a smartphone-compatible CO monitor. This study explores two different lengths of contingency management (6 weeks vs 12 weeks) for this population using a smartphone app and remote CO sensor to monitor urges to smoke, stressors, smoking behaviors, and to validate abstinence daily throughout the intervention. The specific aims of the study are to:

1. Explore the feasibility of conducting a randomized controlled trial to compare the effect of two different contingency structures ($20 or £15 weekly incentives for 6 weeks vs 12 weeks) vs control (no incentives) on smoking abstinence.
2. Explore the feasibility of remote CO testing using a smartphone-based CO sensor and mobile app to validate abstinence.
3. Gather preliminary evidence and estimates of recruitment and eligibility rates, loss to follow-up, as well as the likely effect size of the intervention on smoking cessation to inform parameters of a large randomized controlled trial.
4. Examine the acceptability of the intervention content, delivery mode, timing, adherence, and maintenance.
5. Explore the potential unintended consequences (e.g., weight, glycemic control, perceived health) of the intervention.

## Methods/design

### Study design

A three-arm, randomized controlled feasibility trial will be conducted in two study sites that include the US (Oklahoma City, Oklahoma, USA) and UK (Greater Glasgow, Scotland, UK). The US site is located at the University of Oklahoma Health Sciences Center (OUHSC) campus within the Tobacco Treatment Research Program that is a part of the Oklahoma Tobacco Research Center. This clinic provides in-person and telephone cessation services to the metropolitan area as well as surrounding towns. The UK site is located at the University of Strathclyde and Stop Smoking Services clinics located in Glasgow and Ayrshire and Arran.

This trial will include a baseline assessment (visit 1), quit date visit that occurs 1 week after the baseline assessment (visit 2), and a third follow-up visit (visit 3) at the end of the 12-week cessation program, or 13 weeks after the baseline visit. The participants will complete 12 total weeks of daily assessments using a smartphone beginning on the quit date (visit 2). The 12-week post-quit follow-up period coincides with the 12 weeks of nicotine replacement therapy (NRT) that participants are eligible to receive as part of their standard smoking cessation services.

### Participant eligibility and recruitment

We aim to recruit 30 current smokers with T2D at each study site (*N* = 60), with 10 in each arm of the study at each location. This sample size is based on our primary feasibility outcomes and sufficient for determining completion of the study protocol and adherence to daily assessments. Eligibility criteria for study enrollment includes adults aged 18 to 75, diagnosed with T2D at least 1 year prior to enrollment, current smoking intensity of at least 5 cigarettes per day, and a self-reported quit attempt in the past 12 months indicating that they had previously been unable to quit. Participants will be excluded from the study if they are unwilling to make a quit attempt through the usual cessation care program or are unwilling or unable to use the smartphone and CO monitor following a brief training session.

Participants will be recruited opportunistically in both the US and the UK through multiple avenues, including T2D clinics using physician referrals and clinic flyers, adult smoking cessation services, diabetes support groups, newspaper and online advertisements, posted and emailed recruitment flyers, posters in health and University settings, recontact of research participants interested in future research, and word of mouth. Interested individuals who meet the inclusion criteria will be given an information pack so they can contact the researcher directly to discuss participation. A follow-up appointment will be made for those who choose to participate where eligibility will be checked and informed consent taken.

In the US, this study will be integrated into the current protocol for standard smoking cessation services at the Oklahoma Tobacco Research Center’s (OTRC) Tobacco Treatment Research Program. Individuals who call directly to the smoking cessation clinic or who are referred to the clinic through electronic medical record referral will be screened for eligibility. Any smoker interested in quitting and eligible for this study will be provided information about the study and the opportunity to participate at their initial smoking cessation appointment.

### Intervention and control groups

Participants will be randomized to one of three groups: (1) a short-term (6 weeks) incentives group, (2) a long-term (12 weeks) incentives group, and (3) a control group with no incentives. Participants in all three study arms will receive the usual smoking cessation care available in each setting. Usual care in both sites will include an in-person or telephone counseling component, selected by the participants, for at least a 6-week period as well as NRT and/or medications for a 12-week period. Due to the limitations of the small feasibility study and the reliance on existing treatment programs for usual care, we will not be able to completely standardize the cessation components in both countries. However, we will explore adjustment for treatment intensity including counseling sessions completed and NRT received during the analysis through multivariable modeling to account for differences in usual care received.

The incentives groups will have the opportunity to earn $20 or £15 per week by providing evidence of smoking abstinence using remote CO monitors. We will validate smoking abstinence via remote biochemical verification through the use of the CoVita iCO™ Smokerlyzer® breath monitor, which is a personal and remote CO sensor that plugs directly into a smartphone [37]. The iCO™ Smokerlyzer® has been demonstrated to be highly reproducible and able to differentiate between smokers and nonsmokers; however, results consistently differ slightly suggesting a need for calibration guidelines [38]. The OTRC has internally evaluated the validity and reliability of the iCO™ Smokerlyzer® integrated into the INSIGHT™ application by testing known concentrations of CO at 5 ppm, 10 ppm, and 20 ppm and found that multiple iCO™ Smokerlyzer® sensors were consistent and accurate enough to differentiate smokers from nonsmokers. Evidence of abstinence each week will be defined as 5 out of 7 days (> 70%) with CO readings of less than 8 parts per million (ppm) submitted through the remote CO sensor. Participants in the short-term incentives program will be eligible to earn weekly incentives for the first 6 weeks of the program, or only during the counseling portion of the usual care program. Participants in the long-term incentives program will be eligible to earn weekly incentives throughout the full duration of the 12-week usual care cessation program, which is the length of time they are eligible to receive NRT as part of usual care. Participants in both incentives groups will be notified weekly about incentives earned throughout the duration of follow-up, and earned incentives for both groups will be distributed in the form of gift cards at the end of the 13 week study (visit 3) following the return of the smartphone. Each weekly incentive is independent and does not require continuous abstinence throughout the entire incentive period. All three groups will be instructed to submit daily assessments for the duration of the 12-week program. Individuals in the control group will be unable to earn incentives and will not be compensated for their participation in the research study due to restrictions of the charity funding source. This is common in the UK and is not anticipated to be a problem. However, participants in the US are often compensated for research. Therefore, individuals in the US control group will be informed that they are eligible to win a tablet computer when they complete the 12-weeks and return the study phone.

### Randomization

Participants in this unblinded study will be randomly allocated to one of the three groups based on block randomization. A random number sequence generated by a computer will be created, which will then be used in the randomization feature in Research Electronic Data Capture (REDCap). After an individual provides informed consent at the baseline visit (visit 1), the participant will be asked to return for the quit date at visit 2 1 week later. Prior to visit 2, a study coordinator without prior knowledge of the sequence will randomize the participant via REDCap, allocate the individual to the selected group, and inform the participant of their assigned group at visit 2. Participants will be informed of their assigned group at the second visit in attempt to maximize the likelihood of continuation in the study for individuals who are assigned to the control group and unable to earn incentives. In addition, the control group participants in the US will be reminded of their 1 in 10 chance of winning a tablet after completing the study. Blinding throughout the duration of the study is not feasible because the notification of each weekly earned incentive is part of the intervention.

### Data collection and outcome measures

Assessments of study measures will be obtained at each of the three in-person visits (Week 1, 2, and 13) as well as through a daily assessment using a smartphone that is provided to each participant (Table 1).

**Table 1:** Detailed data collection schedule and measurement items

		**Study period**			
		**Enrollment (-t**_**1**_**)**	**Allocation (0)**	**Post-allocation (t**_**1**_**-t**_**12**_**)**	**Close-out (t**_**13**_**)**
	**Assessment items (#)**	**Visit 1 (baseline, week 1)**	**Visit 2 (quit date, week 2)**	**Daily assessment (week 2 to 13)**	**Visit 3 (week 13)**
**Enrollment**					
Eligibility screen		X			
Informed consent		X			
Allocation			X		
**Interventions**					
Short term incentives (6 weeks)		X	X	X	X
Long term incentives (12 weeks)		X	X	X	X
No incentives (control)		X	X	X	X
**Assessments**					
Biological/anthropometric measures	3	X	X		X
Demographic/background information questionnaire	10	X			
Tobacco history questionnaire	17	X			
Heaviness of smoking index	2	X			
Wisconsin smoking withdrawal scale—craving (past week)	4	X			
Self-efficacy scale/confidence	9	X			
Motivation to quit	3	X			
Diabetes questionnaire	5	X			
Diabetes self-management questionnaire	16	X			X
Consideration of future consequences scale	12	X			
Daily stress and anxiety	5			X	
Daily urges to smoke	4			X	
Daily mood and physical symptoms scale	12			X	
Daily self-efficacy/confidence/motivation	4			X	
Daily tobacco and medication use	7			X	
Daily diabetes and health questions	6			X	
Daily CO reading (iCO™ Smokerlyzer®)	1			X	
Study participation exit questionnaire	10				X

#### Assessments at visits 1, 2, and 3

At all three in-person visits that will occur in the treatment clinics, biological/anthropometric measures will be collected by the research coordinator and entered into a secure REDCap database. These measures include height, weight, and tobacco use status measured through expired CO using a Vitalograph Breath CO monitor. At visits 1 and 3, additional measures will be obtained through self-administered surveys in REDCap using a tablet. Participants will complete 10 items related to demographics, which include sex, age, race/ethnicity, marital status, education, employment status, income, and the status of whether the participant lives with a smoker, has an active mobile phone, and if it is a smartphone. We developed a tobacco use history questionnaire that will capture 17 items related to current and past product, dose, and duration of use of tobacco, quit attempt history, perceptions of harm, comorbidities, and medication use. To assess dependence, participants will complete two items from the heaviness of smoking index [39] as well as 4 items from the Wisconsin smoking withdrawal scale for craving [40], both of which have been shown to be valid and reliable. We will use a 9-item self-efficacy scale/confidence to reflect the confidence that they can cope with high-risk situations without relapsing as well as 3 items assessing motivation to quit [41]. In addition to tobacco-related questions, participants will respond to diabetes-related questionnaires, which include a 5-item section related to age of diagnosis, diabetes control, and glycated hemoglobin and a 16-item diabetes self-management questionnaire assessing self-care activities related to diabetes [42]. Finally, we will collect measures of delayed gratification using the 12-item consideration of future consequences scale [43]. Table 1 outlines the assessments and number of items during the in-person visits.

#### Daily assessments

Beginning at the quit date at week 1 throughout the duration of the 12-week intervention, we will use mobile-based daily assessments to validate abstinence and capture additional data related to stress and anxiety, urges to smoke, mood and physical symptoms, self-efficacy and confidence, motivation, tobacco and medication use, and diabetes information (Table 1). Daily assessments will be collected through the INSIGHT™ mobile health platform developed by OTRC [44]. We will purchase a Samsung Galaxy S7 phone in the US and a Samsung J3 Prime phone in the UK along with a 3-month data plan for each participant. Each participant will also receive a CoVita iCO™ Smokerlyzer® sensor to use throughout the study period. Using a smartphone application platform developed by the OUHSC OTRC mHealth Core, we programmed an app to collect our specific daily measures. The app will be installed onto the study phones and participants will be prompted through an evening alarm to submit daily CO readings (in ppm) and complete the daily assessment throughout the study period. The OUHSC mHealth Core has integrated the CO sensor into the platform with step-by-step instructions within the app that will collect assessment data so that participants will submit all measures in one cohesive assessment.

The daily assessment, estimated to take between 5 and 10 min, will begin with the CO reading followed by 5 items related to daily stress, which include the validated 4-item perceived stress scale [45]; 4 items assessing daily urges to smoke; the validated 12-item mood and physical symptoms scale [46]; 4 items related to self-efficacy, confidence, and motivation to quit; tobacco use, including products used, amount, and cessation medications; and 6 items related to diabetes management, which includes self-rated health, diabetes control, healthy food choices, and physical activity. Study coordinators will monitor compliance to daily assessments and will reach out to participants who quit completing assessments to encourage them to continue completing assessments.

#### Exit surveys and interviews

At visit 3, participants will complete a brief exit survey that includes 10 questions related to their experience in the study. Participants will be asked to rate the difficulty or ease of using the smartphone, the app, and the CO device and about their perception of accuracy of the CO readings. They will be asked about reasons for missing daily assessments, the difficulty or ease of this quit attempt, whether payments may have helped them or could help others to quit, and if they were honest and completed the assessments themselves as instructed. The qualitative interviews will use semi-structured topic guides to gather rich and in-depth information related to the participants’ motivation to take part in the study, their attitudes towards financial incentives, their experiences in the study submitting data, their overall quit attempt, any perceived impact on their diabetes, and suggestions for improvements to the study. The interviews will be carried out in-person or over the telephone according to the participant’s preference and every participant will be approached for recruitment for an interview. The interviews will be audio-recorded and transcribed verbatim.

#### Feasibility outcome measures

As this is a feasibility study, our primary outcomes will be the completion of the study protocol and proportion of daily assessments completed. Our secondary outcomes for the feasibility study will be monthly recruitment rates and participants’ acceptability of study procedures as measured in the exit survey and qualitatively in the exit interviews. Although this pilot study is underpowered to detect differences in cessation between groups, we will also measure and explore differences in the outcomes that would be used in a larger randomized controlled trial: continuous abstinence at 12 weeks, time to relapse, and cigarettes per day throughout the study period.

### Study procedures

At visit 1, the study coordinator will demonstrate the use of the smartphone and remote CO device and answer any questions related to the study procedures. After obtaining informed written consent, the study coordinator will measure height, weight, and CO and the participant will complete the baseline assessments using a REDCap survey on a tablet. At visit 2, 1 week after baseline, the participant will return to the clinic on their quit date. A study coordinator will provide instructions to the participant based on their random group assignment and will provide the participant with their smartphone and CO monitor. The participant will receive written instructions and training on how to use the smartphone and respond to the daily assessments. Participants will receive a 300-min unlimited data and text phone plan and will be permitted to use the phone for calls, text messages, and internet during the 12-week follow-up period to encourage participants to ensure the phone is charged and available at the time of each assessment. This will also be an incentive to submitting data since we are unable to compensate participants for submitting data. Participants will be asked on their preferred method of contact, which includes calls or text messages on their study phone or personal phone. A study coordinator will monitor their data weekly and contact participants throughout the 12-week daily assessment follow-up period to ensure that the phone is working and to answer any questions. Participants who enroll in the study at the baseline visit who miss their quit date and, therefore, do not receive a smartphone or CO monitor will be involuntarily withdrawn from the study if they are not able to reschedule within 1 week. Participants who wish to withdraw from the study at any time will be asked to return the smartphone and will be voluntarily withdrawn at the time of the request. Participants who withdraw from the study are offered the opportunity to provide feedback on the study prior to exiting. At visit 3, participants will complete the exit survey and exit interview and return the study phone and CO device to the study personnel.

### Statistical methods

As this is a feasibility study, we will first examine our primary outcomes by calculating loss to follow-up rates for the follow-up visits and the proportion of daily assessments completed per participant for each study arm. We will also examine monthly recruitment and enrollment rates, and evaluate the acceptability of the program using the exit survey items. Although we are underpowered to test differences, we will explore the feasibility of our study protocol and data completeness by conducting statistical analyses similar to that of a larger trial. We will initially conduct range checks for data values to assess data quality. We will calculate descriptive statistics and compare baseline characteristics between arms to ensure adequate randomization between groups. All questionnaire items will be scored using methodology described in the literature. We will calculate means and standard deviations for continuous measures and frequencies for categorical measures. We will use time-to-event analysis to explore time to relapse and/or time to discontinuing the daily assessments. Smoking cessation assessment data and ecological momentary assessment data will be measured repeatedly and will therefore be correlated within subjects. Thus, our analytic approach will include generalized linear mixed model regression analysis. This analysis approach can handle normal and non-normal outcomes, such as dichotomous smoking status variables, different variance functions, as well as unbalanced designs where the number of repeated observations varies across individuals. Analyses will be conducted using both intention-to-treat and per-protocol approaches. All preliminary results will be interpreted with caution and treated as preliminary due to small numbers in each arm of this feasibility study. For the qualitative components of this study, we will use the transcribed interviews to develop codes and we will use thematic analysis to determine the feasibility and acceptability of this study protocol.

### Ethical considerations and dissemination policy

This study protocol outlined in Table 2 has been reviewed and approved by the University of Oklahoma Health Sciences Center Institutional Review Board (IRB# 8652) as well as the NHS Welsh Research Ethics Committee, (IRAS ID 231008). Each participant will provide informed written consent prior to enrollment. Participants will be assured that their participation is voluntary and that they have the right to withdraw at any time. They will also be informed that choosing not to participate will not affect their receipt of standard smoking cessation services. Anonymized and aggregated findings will be presented at conference and submitted for publication in academic peer-reviewed journals.

**Table 2:** World Health Organization trial registration data set

Data category	Information
Primary registry and trial identifying number	ClinicalTrials.gov, NCT03527667
Date of registration in primary registry	May 4, 2018
Secondary identifying numbers	N/A
Source(s) of monetary or material support	Cancer Research UK PO Box 1561 Oxford OX4 9GZ Phone: 0300 123 1022
Primary sponsor	Cancer Research UK
Secondary sponsor(s)	Stephenson Cancer Center
Contact for public queries	Sydney Martinez, PhD (405)271-2229, sydney-martinez@ouhsc.edu
Contact for scientific queries	Sydney Martinez, PhD (405)271-2229, sydney-martinez@ouhsc.edu
Public title	Incentivized smoking cessation for diabetics
Scientific title	Incentivized smoking cessation for tobacco treatment-resistant diabetics
Countries of recruitment	United States, United Kingdom
Health condition(s) or problem(s) studied	Diabetes, smoking
Intervention(s)	6-weeks vs 12-weeks of financial incentives for proof of abstinence
Key inclusion and exclusion criteria	Inclusion criteria—ages 18 to 75, type 2 diabetes diagnosis greater than 1 year prior to enrollment, currently smoking at least 5 cigarettes per day, self-reported quit attempt in past 12 months Exclusion criteria—unwilling to make a quit attempt through usual care, unable to use smartphone and iCO™ Smokerlyzer® following training session
Study type	Interventional (clinical trial) Allocation: randomized Exploratory
Date of first enrolment	June 22, 2018
Target sample size	60
Recruitment status	Recruiting
Primary outcome(s)	Proportion of CO readings completed
Key secondary outcomes	Quit outcomes; cigarettes per day

## Discussion

Diabetes management is complex, and individuals with T2D are often encouraged to change multiple behaviors, such as diet, exercise, glucose monitoring, and medications. Smoking cessation is an important behavior modification that is recommended for individuals with T2D, but cessation often competes with other lifestyle modifications that take precedence and individuals with T2D often struggle to quit. This feasibility study aims to explore novel ways of improving the smoking cessation outcomes among individuals with T2D through contingency management. As this is a feasibility study, we will explore the ability to recruit smokers with T2D who are interested in quitting and measure their engagement in the program, particularly related to the use of the remote biochemical verification using a smartphone app. In addition, we will evaluate the intervention content, study procedures, data collection methods, and follow-up and will qualitatively assess the participants’ acceptability of the program, including the duration of the daily assessments. While it is unclear what duration of daily assessments will be acceptable to this study population, other studies have used daily assessments for a wide range of durations with success [47]. We have limited our daily assessment to one brief assessment per day at the time of the participant’s choice to minimize burden throughout the 12-week period.

As this study was developed through a unique multidisciplinary collaboration between investigators in the US and UK, this protocol was designed to account for cultural differences between the two sites. We included subtle differences across sites in regards to recruitment methods, questionnaire items, the cell phone and data plans that will be provided, the amount of incentives that can be earned, and the integration of our study protocol into the local standard cessation services. In designing the protocol, we discovered cultural differences in the expectation of compensation for the participation in research. In the US site, community members are often provided compensation for engaging in research studies, whereas participants in the UK are not routinely offered compensation for research carried out in publicly funded healthcare settings. To account for differences in motivation to participate in research that might pose potential difficulties in recruitment or the engagement of controls in the US, we added a chance to win a tablet for the control group in the US site. Although there is a concern that there might be differential drop out in the control group due to the inability to earn incentives or be compensated for submitting data, this is a feasibility study and this will be evaluated as part of acceptability and feasibility measures. The protocol also included two similar versions of the study assessments that accounted for cultural and language differences, with subtle variations in the response options on survey items, such as race and ethnicity, employment and income.

Overall, this unique international and multidisciplinary protocol will provide an opportunity to examine the feasibility of using mobile technology and financial incentives to increase the success in quitting smoking among a population that faces many critical lifestyle changes that undermine motivation and perceived capability for quitting. With T2D and other chronic conditions on the rise, it is important to explore new ways of addressing smoking cessation for patients with multiple behavior change priorities.

## Trial status

Protocol version: 1, May 4, 2018

Recruitment began June 1, 2018

Anticipated recruitment end date: May 31, 2019

## Data Availability

Not applicable

## Abbreviations

T2D: Type 2 diabetes
US: United States
UK: United Kingdom
CO: Carbon monoxide
mHealth: Mobile health
App: Application
OUHSC: University of Oklahoma Health Sciences Center
NRT: Nicotine replacement therapy
Ppm: Parts per million
REDCap: Research electronic data capture

## References

[1] Sigmon SC, Miller ME, Meyer AC (2016). Financial incentives to promote extended smoking abstinence in opioid-maintained patients: a randomized trial. Addiction..

[2] Centers for Disease Control and Prevention. National diabetes statistics report: estimates of diabetes and its burden in the United States. Atlanta, GA: Centers for Disease Control and Prevention; 2017. US Dep Heal Hum Serv. 2017;(Cdc):2009–2012. 10.1177/1527154408322560.

[3] Menke A, Casagrande S, Geiss L, Cowie CC (2015). Prevalence of and trends in diabetes among adults in the United States, 1988-2012. JAMA - J Am Med Assoc..

[4] US Department of Health and Human Services. The health consequences of smoking-50 years of progress: a report of the surgeon general, executive summary. A Rep Surg Gen. 2014. NBK179276.

[5] Huang ES, Basu A, O’Grady M, Capretta JC (2009). Projecting the future diabetes population size and related costs for the U.S. Diabetes Care..

[6] Tonstad S (2009). Cigarette smoking, smoking cessation, and diabetes. Diabetes Res Clin Pract..

[7] Haire-Joshu D, Glasgow RE, Tibbs TL (1999). Smoking and diabetes. Diabetes Care..

[8] Eliasson B (2003). Cigarette smoking and diabetes. Prog Cardiovasc Dis..

[9] Iino K, Iwase M, Tsutsu N, Iida M (2004). Smoking cessation and glycaemic control in type 2 diabetic patients. Diabetes Obes Metab..

[10] Sherman J (2005). The impact of smoking and quitting smoking on patients with diabetes. Diabetes Spectr..

[11] Willi C, Bodenmann P, Ghali WA, Faris PD, Cornuz J (2007). Active smoking and the risk of type 2 diabetes: a systematic review and meta-analysis. JAMA..

[12] Bajaj M (2012). Nicotine and insulin resistance: when the smoke clears. Diabetes..

[13] Giovannucci E, Harlan DM, Archer MC, Bergenstal RM. Diabetes and cancer: a consensus report. a Cancer J Clin. 2010;60(4):207-221. 10.3322/caac.20078.Available. 10.3322/caac.20078 20554718

[14] International Agency for Research on Cancer. World cancer report 2008. Cancer Control. 2008;199:512. 10.1016/j.cma.2010.02.010.

[15] Liu X, Ji J, Sundquist K, Sundquist J, Hemminki K (2012). The impact of type 2 diabetes mellitus on cancer-specific survival: a follow-up study in Sweden. Cancer..

[16] Mayor S (2005). High glucose and diabetes increase cancer risk. Lancet Oncol..

[17] Hex N, Bartlett C, Wright D, Taylor M, Varley D (2012). Estimating the current and future costs of type1 and type2 diabetes in the UK, including direct health costs and indirect societal and productivity costs. Diabet Med..

[18] American Diabetes Association. Lifestyle management: standards of medical care in diabetes—2018. Vol 41.; 2018. 10.2337/dc18-S004. 10.2337/dc18-S004 29222375

[19] Nagrebetsky A, Brettell R, Roberts N, Farmer A (2014). Smoking cessation in adults with diabetes: a systematic review and meta-analysis of data from randomised controlled trials. BMJ Open..

[20] Higgins ST, Washio Y, Heil SH, et al. Financial incentives for smoking cessation among pregnant and newly postpartum women. Prev Med (Baltim). 2012;55(SUPPL.):S33-S40. 10.1016/j.ypmed.2011.12.016. 10.1016/j.ypmed.2011.12.016 PMC3399924 22227223

[21] Ledgerwood DM, Arfken CL, Petry NM, Alessi SM (2014). Prize contingency management for smoking cessation: a randomized trial. Drug Alcohol Depend..

[22] Volpp K, Levy A, Asch D (2006). A randomized controlled trial of financial incentives for smoking cessation. Cancer Epidemiol Biomarkers Prev..

[23] Ondersma SJ, Svikis DS, Lam PK, Connors-Burge VS, Ledgerwood DM, Hopper JA (2012). A randomized trial of computer-delivered brief intervention and low-intensity contingency management for smoking during pregnancy. Nicotine Tob Res..

[24] Hertzberg JS, Carpenter VL, Kirby AC (2013). Mobile contingency management as an adjunctive smoking cessation treatment for smokers with posttraumatic stress disorder. Nicotine Tob Res..

[25] Businelle MS, Kendzor DE, Kesh A (2014). Small financial incentives increase smoking cessation in homeless smokers: a pilot study. Addict Behav..

[26] Cahill K, Hartmann-Boyce J, Perera R (2015). Incentives for smoking cessation (Review). Cochrane Database Syst Rev..

[27] Dallery J, Raiff BR, Grabinski MJ (2013). Internet-based contingency management to promote smoking cessation: a randomized controlled study. J Appl Behav Anal..

[28] Dallery J, Raiff BR, Kim SJ, Marsch LA, Stitzer M, Grabinski MJ. Nationwide access to an internet-based contingency management intervention to promote smoking cessation: a randomized controlled trial. Addiction. 2016:875–83. 10.1111/add.13715. 10.1111/add.13715 PMC5382065 27923264

[29] Volpp KG, Levy AG, Asch DA (2006). A randomized controlled trial of financial incentives for smoking cessation. Cancer Epidemiol Biomarkers Prev..

[30] Crowley T, Macdonald M, Walter M. Behavioral anti-smoking trial in chronic obstructive pulmonary disease patients. Psychopharmacology (Berl). 1995;119(2 PG-193-204):193-204. NS -. . 10.1007/BF02246161 7659767

[31] Walter K, NM P. (2015). Patients with diabetes respond well to contingency management treatment targeting alcohol and substance use. Psychol Heal Med..

[32] Raiff BR, Dallery J (2010). Internet-based contingency management to improve adherence with blood glucose testing recommendations for teens with type 1 diabetes. J Appl Behav Anal..

[33] Raiff BR, Jarvis BP, Dallery J (2016). Text-message reminders plus incentives increase adherence to antidiabetic medication in adults with type 2 diabetes. J Appl Behav Anal..

[34] Dallery J (2012). Raiff BR. Innovations to promote smoking cessation..

[35] Zapata BC, Fernández-Alemán JL, Idri A, Toval A (2015). Empirical studies on usability of mHealth apps: a systematic literature review. J Med Syst..

[36] Vinci C, Haslam A, Lam CY, Kumar S, Wetter DW (2018). The use of ambulatory assessment in smoking cessation. Addict Behav..

[37] CoVita. iCO Smokerlyzer, the world’s first personal Smokerlyzer breath CO monitor for use with your smartphone and tablet. https://www.covita.net/ico-overview/. Published 2020. Accessed March 1, 2020.

[38] Wong HY, Subramaniyan M, Bullen C, Amer Siddiq AN, Danaee M, Yee A. The mobile-phone-based iCOTM Smokerlyzer®: comparison with the piCO+ Smokerlyzer® among smokers undergoing methadone-maintained therapy. Tob Induc Dis. 2019;17(September):1-5. 10.18332/tid/111355. 10.18332/tid/111355 PMC6770613 31582954

[39] Borland R, Yong HH, O’Connor RJ, Hyland A, Thompson ME (2010). The reliability and predictive validity of the heaviness of smoking index and its two components: findings from the International Tobacco Control Four Country Study. Nicotine Tob Res..

[40] Adkison SE, Rees VW, Bansal-Travers M, Hatsukami DK, O’Connor RJ (2016). Psychometric characteristics of the brief wisconsin inventory of smoking dependence motives among a nonclinical sample of smokers. Nicotine Tob Res..

[41] Velicer W, Diclemente C, Rossi J, Prochaska J (1990). Relapse situations and self-efficacy: an integrative model. Addict Behav..

[42] Schmitt A, Gahr A, Hermanns N, Kulzer B, Huber J, Haak T. The diabetes self-management questionnaire ( DSMQ ): development and evaluation of an instrument to assess diabetes self-care activities associated with glycaemic control. 2013:1-14. 10.1186/1477-7525-11-138 PMC3751743 23937988

[43] Strathman A, Gleicher F, Boninger D, Edwards C (2013). Considerations of future consequences (CFC scale).

[44] Mobile Health Technology (mHealth) Shared Resource. Insight Platform.Oklahoma Tobacco Research Center. https://otrc.stephensoncancercenter.org/Mobile-Health-Technology/Insight-Platform. 2016. Accessed 1 Mar 2020.

[45] Warttig SL, Forshaw MJ, South J, White AK (2013). New, normative, English-sample data for the short form perceived stress scale (PSS-4). J Health Psychol..

[46] West R, Hajek P (2004). Evaluation of the mood and physical symptoms scale (MPSS) to assess cigarette withdrawal. Psychopharmacology (Berl)..

[47] Heron KE, Smyth JM (2010). Ecological momentary interventions: incorporating mobile technology into psychosocial and health behaviour treatments. Br J Health Psychol..

